# The Top 100 Most Cited Articles Published in Dentistry: 2020 Update

**DOI:** 10.3390/healthcare9030356

**Published:** 2021-03-21

**Authors:** Faris Yahya Asiri, Estie Kruger, Marc Tennant

**Affiliations:** 1 Department of Preventive Dentistry, College of Dentistry, King Faisal University, Al-Ahsa 31982, Saudi Arabia; 2 International Research Collaboration—Oral Health and Equity, School of Human Sciences, Faculty of Science, The University of Western Australia, Perth, WA 6009, Australia; estie.kruger@uwa.edu.au (E.K.); marc.tennant@uwa.edu.au (M.T.)

**Keywords:** bibliometric analysis, citation analysis, dentistry, most cited

## Abstract

This bibliometric review is aimed to analyze the top 100 most-cited publications in dentistry and to compare its outcomes. A literature search was performed using Elsevier’s Scopus, without any restriction of language, publication year, or study design. Of 336,381 articles, the top 100 were included based on their citation count, which ranged from 638 to 4728 citations (Feijoo et al., 326 to 2050). The most productive decade was the 2000s, with 40 articles on the list (Feijoo et al., 1980s: 26). Marx RE (7%) was the major contributor in this study (Feijoo et al., Socransky SS: 9%), and almost half (48%) of articles were from the USA. Of the top 100 articles, 26% focused on periodontology (Feijoo et al., periodontology: 43%), while 17% of the total were published in the Journal of Dental Research (Feijoo et al., Journal of Clinical Periodontology: 20%). Most of the publications were narrative reviews/expert opinion (36%), (Feijoo et al., case series: 22%), and were within the evidence level V (64%) (Feijoo et al., 54%). The citation count that a paper secures is not necessarily a reflection of research’s quality, however, the current analysis provides the latest citation trends in dentistry.

## 1. Introduction

As a science, dentistry has reached a high maturity level in recent decades [1]. In academia, journals play a crucial role by disseminating technical and scholarly work, peer-review and evaluating research, archiving such research, and providing a foundation for scholarly credits [2]. In 2004, Olk and Griffith stated that journals serve as the primary source of knowledge in a particular specialty. They argued that the boundaries of a given discipline are pushed by scholars, however, journals are essential to advance the main body of knowledge [3]. The American Journal of Dental Science, the world’s first dental journal, began its publication in 1839 [4]. Since then, journals in dentistry have been performing as a mode of communication and source of knowledge within the dental community and other related fields. Hence, valid and reliable tools are necessary to analyze and document several changes that may occur in the lifetime of a single academic journal or group of journals [2].

Citations are potential indicators of a publication’s impact in this expanding scientific literary environment [5]. A citation is an alphanumeric expression that acknowledges a particular subject’s contribution to others’ research [6,7]. Citation analysis is a bibliometric method to identify articles with the greatest impact on research and the clinical community in a given discipline [8], providing the foundation for developing new research lines, techniques, and theories. This method has been adopted in different dentistry subfields including endodontics, orthodontics, periodontology, implant dentistry, prosthodontics, oral and maxillofacial surgery, dental traumatology, dental caries, oral squamous cell carcinoma, oral submucous fibrosis, oral leukoplakia, cleft lip and palate, and medication-related osteonecrosis of the jaw (MRONJ) [9,10,11,12,13,14,15,16,17,18,19,20,21,22]. The definition of “classic article” has been a controversial topic across disciplines, and the most commonly suggested criterion has been the securing of a certain citation count, for instance, at least 400 citations [8,23,24]. However, a publication having accomplished 100 or more citations can also be termed as a “classic publication,” depending upon the field under consideration, such as dentistry [25].

This bibliometric review aimed to identify and analyze the scientific activity of dental sciences up to 2020. The Elsevier’s Scopus database was utilized to accomplish three specific objectives: (a) characterize the dental research in association with output, impact, geographic origin, authorship, topic, methodology design, and evidence level; (b) thematically categorizing research in dental areas, analyzing their interactions and evaluating their up-to-date trends; (c) assess any changes in citation trends of dentistry articles when compared with a similar, but much earlier study, published by Feijoo et al. [9] in 2014.

## 2. Results

### 2.1. Citation Count, Citation Density, and Current Citation Index

The primary characteristics of the top 100 most-cited articles in dentistry are shown in Supplementary Table S1. Overall, the 100 most-cited articles published in dentistry journals achieved a total of 113,482 (Scopus) and 214,642 (Google Scholar) citations; with the citation count varying between 638 and 4728 (Scopus), and 138 and 8281 (Google Scholar). According to Scopus, 33 articles exceeded 1000 citations; with 33 articles securing more than 2000 citations as per Google Scholar. The most cited article, with a total of 4728 (Scopus), 8281 (Google Scholar) citations, was as a clinical trial titled “Periodontal Disease in Pregnancy II. Correlation between Oral Hygiene and Periodontal Condition” [26], and was published in the *Acta Odontologica Scandinavica*. Its citation density was 84, with the current citation index of 269. The second most cited article, with a total of 4062 (Scopus), 7873 (Google Scholar) citations, was similar to the first article, but was published one year earlier titled “Periodontal disease in pregnancy I. Prevalence and severity” [27], and was also published in the *Acta Odontologica Scandinavica*. Its citation density was 71, with the current citation index of 232. The third most cited article, with a total of 3392 (Scopus), 6257 (Google Scholar) citations, was also a clinical trial titled “A 15-year study of osseointegrated implants in the treatment of the edentulous jaw” [28] and was published in the International Journal of Oral and Maxillofacial Surgery. Its citation density was 117, with the current citation index of 96.

As per citation density, a review by Guo and DiPietro [29] has the highest score, i.e., 186. In the second rank, with a citation density of 181, is an article related to the category of classification or tools for assessing results [30]. The third-ranked article (citation density of 167) is a position paper by Ruggiero et al. [31]. According to the current citation index 2020, the top-ranked article was a review published in 2010, securing 345 citations [29]. The second-ranked article was a recommendation paper related to the category of classification or tools for assessing results written by Schiffman et al. [30] in 2014, with 299 citations. The third-ranked article was a clinical trial by Sillness and Löe, which counted 269 citations [26].

**Figure 1:**
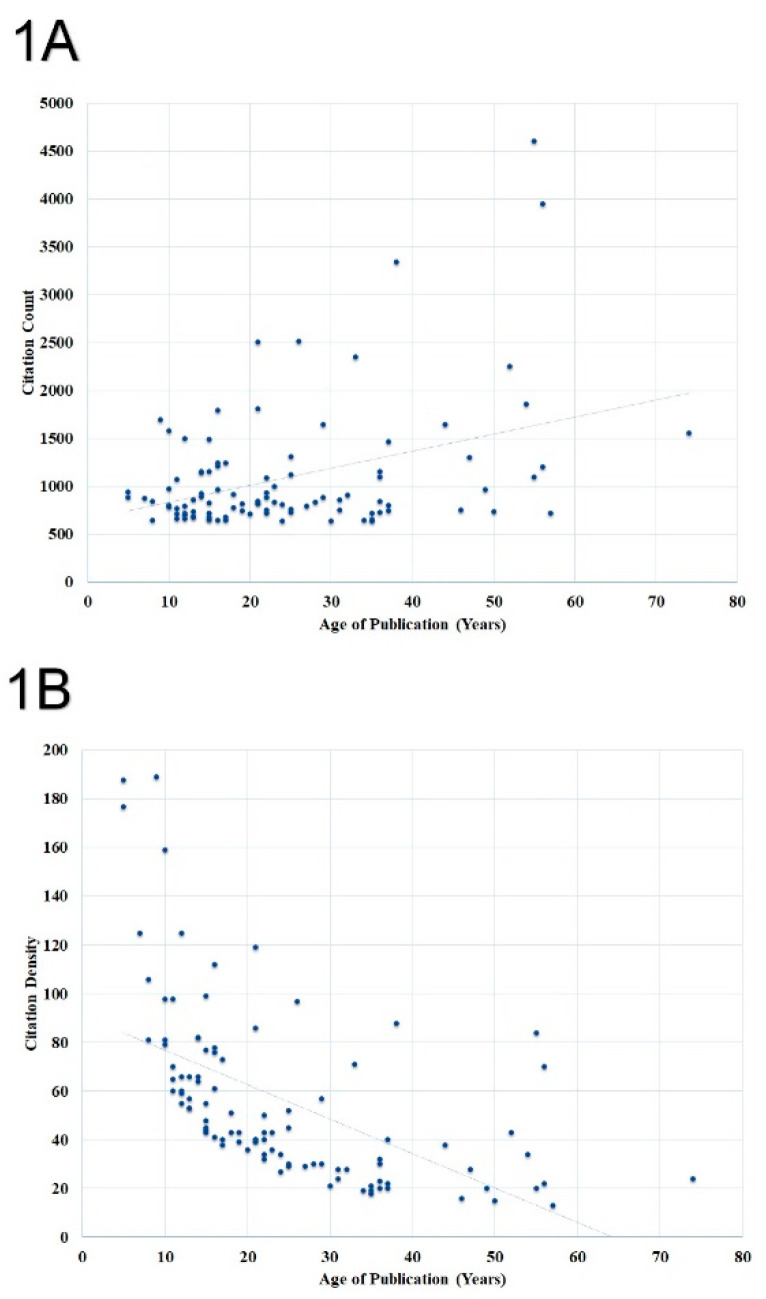
Association of (**A**) citation count and (**B**) citation density with the age of publication. The dot represents individual publication and line represents trendline.

According to the Shapiro–Wilk test, the distribution of data regarding citation count, citation density, and article age was not normal (*p* < 0.01). A significant trend towards a higher citation count with article age was observed (r = 0.832, *p* < 0.01) (Figure 1A). However, a non-significant trend towards an increased citation density with the age of publication was observed (r = 0.176, *p* = 0.129) (Figure 1B).

### 2.2. Distribution by Year

**Figure 2:**
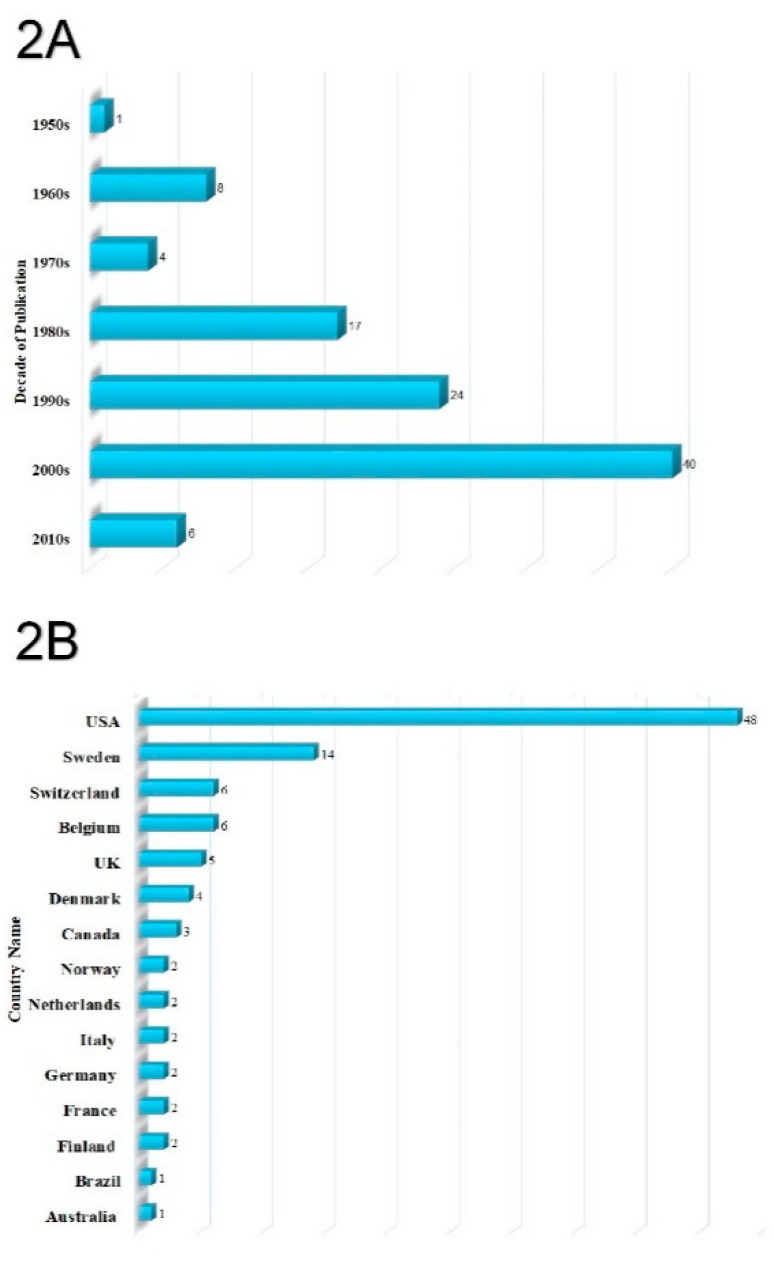
(**A**) Citation analysis of the top 100 most-cited articles over the decades. (**B**) The contribution of countries to the top 100 articles.

The top 100 most-cited articles were published between 1955 [32] and 2014 [30,31]. The most prolific year in terms of publications was 2004, with seven publications, followed by 1997, 1998, 2003, and 2007 with five articles each. The year with most citations was 1998, with 6829 citations, followed by 2004 and 2003, with 6190 and 5879 citations, differently. The decade with most publications (*n* = 40) and citations (*n* = 35,743) was the 2000s (Figure 2A).

### 2.3. Contribution of Countries

**Figure 3:**
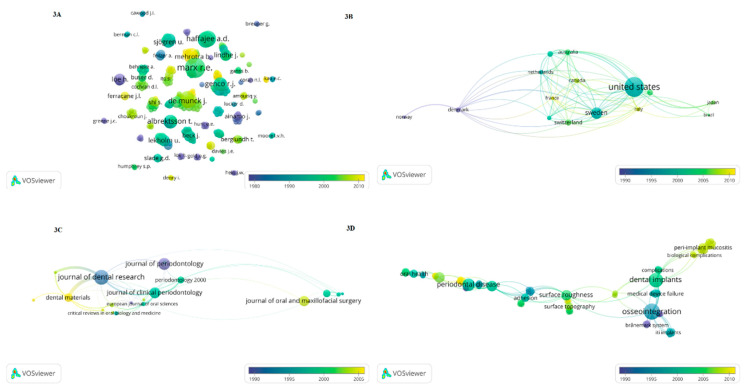
(**A**) Network visualization per author (Elsevier’s Scopus data 1980–2010). (**B**) Network visualization per count (Elsevier’s Scopus data 1990–2010). (**C**) Network visualization per journal (Elsevier’s Scopus data 1990–2010). (**D**) Network visualization per author keywords (Elsevier’s Scopus data 1990–2010).

The top 100 most-cited publications originated from 15 countries, including Australia, Belgium, Brazil, Canada, Denmark, Finland, France, Germany, Italy, Netherlands, Norway, Sweden, Switzerland, the United Kingdom (UK), and the United States of America (USA) (Figure 2B and Figure 3A). According to the number of publications, most of the articles originated from the United States of America (*n* = 48), followed by Sweden (*n* = 14), Belgium (*n* = 6), Switzerland (*n* = 6), UK (*n* = 5), Denmark (*n* = 4), Canada (*n* = 3), Finland (*n* = 2), France (*n* = 2), Germany (*n* = 2), Italy (*n* = 2), Netherlands (*n* = 2), Norway (*n* = 2), Australia (*n* = 1), and Brazil (*n* = 1).

### 2.4. Contribution of Authors

A total of 264 authors contributed to the top 100 most-cited articles. Many of the articles (*n* = 84) had between one and six authors, but publications with two authors were the most common (*n* = 27). The majority of the contributions were made by Marx RE (*n* = 7, 8230 citations), followed by Löe H (*n* = 4, 12,668), Lekholm U (*n* = 4, 6654), Haffajee AD (*n* = 4, 5313), Socransky SS (*n* = 4, 4843), Albrektsson T (*n* = 4, 4658), De Munck J (*n* = 4, 3772), and Genco RJ (*n* = 4, 3014) (Table 1 and Figure 3B).

### 2.5. Journal of Publication

Overall, the top 100 most-cited articles in dentistry were published in both specialized and comprehensive periodicals (*n* = 31) (Table 2 and Figure 3C). The journal with the greatest number of publications was the *Journal of Dental Research* (*n* = 17, 17,836 citations), followed by *Journal of Periodontology* (*n* = 11, 12,141), *Journal of Clinical Periodontology* (*n* = 9, 8461), *Journal of Oral and Maxillofacial Surgery* (*n* = 8, 8873), *Dental Materials* (*n* = 7, 6220), *Journal of Endodontics* (*n* = 5, 3927), and *Periodontology 2000* (*n* = 4, 3391).

A statistically non-significant trend (*p* = 0.204) was observed between a journal age and the number of articles published in that journal. However, a statistically significant trend (*p* < 0.05) was observed between the impact factor of the journal and the number of articles published in that journal.

### 2.6. Field of Interest

For the 100 most-cited articles, the field of interest for the majority were related to Periodontology (*n* = 26, 32,410 citations), adhesive restorations (*n* = 14, 11,915), implantology (*n* = 13, 15,592), oral medicine/pathology (*n* = 12, 12,785), endodontics (*n* = 8, 5936), oral hygiene (*n* = 8, 10,643), bone morphology/histology (*n* = 7, 6943), oral biology/morphology (*n* = 4, 5862), regenerative dentistry (*n* = 2, 2228), orthodontics (*n* = 2, 1814), saliva/biochemistry (*n* = 1, 917), pain dysfunction/orofacial pain syndrome (*n* = 1, 941), dental radiology (*n* = 1, 735), and behavior management (*n* = 1, 735) (Table 3).

### 2.7. Methodological Design of the Publication

The most common methodological design in the top 100 publications was literature review/expert opinion (*n* = 36, 34,628 citations), followed by clinical trial (*n* = 24, 34,296), classification or tool for assessing results (*n* = 11, 14,072), systematic review/meta-analysis (*n* = 9, 6627), in vitro study (*n* = 7, 7561), animal study (*n* = 4, 4063), new material or technique (*n* = 4, 3048), cohort study (*n* = 2, 1879), consensus report (*n* = 1, 767), randomized controlled trial (*n* = 1, 717), and letter to editor (*n* = 1, 1798) (Table 3).

### 2.8. Evidence Level of Publication

The top 100 most-cited publications could be classified into all evidence levels (Table 3). Most of the articles were within evidence level V (*n* = 64, 65,937 citations), followed by evidence level IV (*n* = 24, 34,296), evidence level 1 (*n* = 9, 6627), evidence level III (*n* = 2, 1879), and evidence level II (*n* = 1, 717). Among these evidence levels, the total citation counts (r = −0.226, *p* = 0.078) and the citation density (r = 0.082, *p* = 0.633) did not vary significantly.

### 2.9. Author Keywords

A total of 538 keywords were identified from the top 100 most-cited articles (Figure 3D). The most frequently used keyword was osseointegration (*n* = 6), followed by dental implants (*n* = 5), periodontal disease (*n* = 4), periodontitis (*n* = 3), review (*n* = 3), surface roughness (*n* = 3), dentin (*n* = 3), epidemiology (*n* = 3), and wound healing (*n* = 3).

### 2.10. Comparison with the Bibliometric Analysis by Feijoo et al.

Table 4 depicts the main differences between the present study and the bibliometric analysis performed by Feijoo et al. [9]. In the current study, for screening and identifying the most-cited articles, the author utilized Scopus as the benchmark database and used Google Scholar to crossmatch the citation data. On the contrary, Feijoo et al. [9] employed the Web of Science as the benchmark database only. A total of 10 bibliometric parameters were evaluated in the current analysis as compared to Feijoo et al. [9] in which 7 bibliometric variables were assessed. For an unknown reason, the journal *Acta Odontologica Scandinavica* was not included in the study by Feijoo et al. [9]. Interestingly, the 1st and 2nd ranked articles in the present analysis were published in the *Acta Odontologica Scandinavica*. In the present analysis, 48 articles present in the study conducted by Feijoo et al. [9], could secure their position. In the present analysis, an increase of almost two-fold in the total citation counts of the top 100 most-cited articles (113,482 citations) was observed as compared to Feijoo et al. [9] (52,635 citations). According to the Web of Science, the range of citation counts in the present study varied between 3 and 4321, as compared to Feijoo et al. [9] in which the range was between 326 and 2050. According to the Web of Science, 4 and 35 articles could secure ≥1000 and ≥500 citations respectively, in the study conducted by Feijoo et al. [9]. However, in the current analysis, 33 and 100 articles secured ≥1000 and ≥500 citations, respectively. The decade with the majority of publications was the 2000s (40%) in the present analysis as compared to the study by Feijoo et al. [9] in which the 1980s was the most productive decade in terms of the number of top-cited articles (26%). In the present study, publications having two authors (27%) were the most common as compared to the study by Feijoo et al. [9] in which single-author papers (25%) were the most frequent. The biggest contribution was made by Marx RE (7%) in the current study, as compared to Feijoo et al. [9] in which Socransky SS made the biggest contribution (9%). The *Journal of Dental Research* (17%) was the most prolific in the current analysis, compared to the *Journal of Clinical Periodontology* (20%) in the Feijoo et al. study [9]. In both the analyses, articles related to periodontology were the most cited ones. In terms of study design, narrative review/expert opinion (36%) was the most commonly cited methodological design in the current study as compared to an analysis by Feijoo et al. [9] in which case series (22%) was the most frequently cited study design. In terms of evidence level of the publications, articles having evidence level V were the most cited in both the studies.

## 3. Discussion

Authors’ bibliometric analysis allows readers to gain historical insight and development of a particular specialty by identifying and analyzing the most-cited publications that could assist researchers in understanding the emerging themes and future trends for a particular discipline [33,34,35]. For instance, the number of citations a publication receives could indicate other researchers’ interest in using the information for their research. Highly cited articles could display a tendency in clinical practice and may therefore be considered to produce greater research and clinical interest in the reported disciplines [36]. Being “most-cited” article reflects its more frequent contribution to the studies published afterward; however, this characteristic alone does not provide sufficient information regarding its current impact and scientific quality, as the main motive of citers in the selection of reference is in establishing the utility within research, rather than scientific quality [37,38,39]. As per the definition of a “classic article”, all the articles included in this study are called “classic articles” [8,22,23].

The accuracy of bibliometric analyses might be negatively influenced by the limitations of the search engine used. Elsevier’s Scopus, Google Scholar, and Clarivate Analytics’ Web of Science may differ quantitatively or qualitatively concerning the citation count of a publication depending upon the discipline of the study [12,16,40], journals [41], and years [42] in which they were published. Additionally, some publications might not be available in all of these search engines [16,25,43,44]. There were several reasons for not selecting either Google Scholar or Web of Science databases as the benchmark for this analysis. For instance, Google Scholar includes citations from non-scholarly publications including dissertations and thesis, conference papers, technical reports, books, and preprints, which may affect the analysis of the most-cited articles when the target is more specific, as in the present study [44]. However, in Web of Science, missing references are a considerable issue [40], which is a likely reason why Buonocore’s highly cited paper [32] in Google Scholar (4367 citations) and Scopus (1560 citations) was so under cited in Web of Science (427 citations). Similarly, Löe’s [45] highly cited article in Google Scholar (4019 citations) and Scopus (2257 citations) received only 3 citations in Web of Science. It is important to note that both the abovementioned articles were present in the Web of Science “All Databases” section, and not in the Web of Science “Core Collection”. One of the several reasons for selecting Scopus as the benchmark database was that it combines the features of PubMed and Web of Science. These combined characteristics enable improved utility for medical literature research and academic requirements (i.e., citation analysis) [43]. Moreover, Scopus is regarded as the largest citation and abstract search engine of peer-reviewed literature. It is devised to aid researchers in not only accessing scientific information but screening literature for analysis [46], and it has been employed in numerous published bibliometric analyses [25,47,48]. In Scopus, citation analysis is faster and includes more publications than that of Web of Science [49]. In a recently performed study for evaluating the accuracy of citation information in Web of Science and Scopus databases, the authors stated that the former database includes 16.7% incorrect references, also called phantom references, 26.7% error in references (i.e., incorrect volume number or publication year), and 55% missing references [44]. Overall, the author thought Scopus to be the better tool for this study as compared to the similar study by Feijoo et al. [9] that employed Web of Science as the benchmark database.

In many bibliometric studies, it was reported that relevant studies were distributed among journals following Bradford’s law [49,50,51]. According to this bibliometric law, a few prolific journals account for a considerable percentage of all publications in a given discipline [52]. The studies published in these core journals are more probable to be referred to most commonly by successive articles [53]. Interestingly, in this study, the journal distribution pattern of the most-cited publications does not completely fit this law, as the list also features journals such as the *Acta Odontologica Scandinavica* and the *Journal of Dental Research*, which are not considered as the specialized journals in the field of periodontics and adhesive restorations respectively but published few of top-cited articles. Hence, the application of this law for conducting bibliometric analysis in some disciplines may cause inaccurate inferences. In this study, a statistically significant association was found between the number of the most-cited articles published in a journal and the impact factor of that journal. This finding is in accordance with the findings of some bibliometric studies [52,53,54,55], but contrary to those of several others [54,56].

As with several “most-cited” publications in dentistry [8,53,54,55,56,57,58], this study reported that most of the most-cited articles in dentistry originated from the United States. This significant contribution can be attributed to a larger scientific population, active researchers, and ample financial resources [10,17,59,60,61]. Additionally, to unparalleled research work, an increased tendency among authors to cite articles originating from the US has been observed [17,62]. It is noteworthy that approximately 47% of the most cited dentistry articles, including the 1st and 2nd, ranked articles in this study, originated from European institutions, despite their small population size. Importantly, a lack of multicenter studies was noticeable, reflecting a need to escalate international collaboration.

Overall, after the US, European countries, including Sweden, Belgium, Switzerland, UK, and Denmark, have been prominent in this list of contributing authors. Additionally, to this study, several other bibliometric analyses have reported that authors from Asia, Africa, and the Middle East, whether being the first or the corresponding author made a negligible contribution to what could be considered a top-cited article [17,60,63,64]. Potential reasons might include language barriers, gaps in conducting research, and professional networking, as well as limited information access [65]. International organizations such as the World Health Organization [WHO] and the United Nations [UN] could play a vital role in bolstering these health care developments.

The particular subject area of the highly cited papers fluctuates from one decade to another. Overall, in the present study, there was a domination of articles related to periodontology, specifically on the topic of microbiology, although other disciplines of dentistry, including adhesive restorations and implantology, have been progressively incorporated. A considerable portion of our analysis comprised of narrative reviews (36%). It might be argued that this category of publication does not follow the concept of reproducible science [66] as a systematic review does [67]. Interestingly, the findings of this study are in opposition to this concept of being a narrative review or systematic review. When compared to the baseline references, randomized controlled trials, a narrative review appeared to secure higher citations than a systematic review. One possible explanation might be that narrative reviews aim to explain the mechanisms of diseases or hypothesis generation; hence, a systematic method to synthesize the evidence in these cases may be irrelevant. Furthermore, as these narrative reviews are authored by the experts in the respective specialty and supported by reputed institutions, readers tend to believe that these articles are not overly sensitive to bias. Nevertheless, in opposition to the previous concerns about the non-reproducibility of narrative reviews, future research is therefore required to explain the extent to which scientific advancement is encouraged through systematic (in comparison with narrative) reviews. Interestingly, the dental journal with the current highest impact factor, *Periodontology 2000*, is focused on publishing narrative reviews. After narrative reviews, clinical trials are the most frequently cited study design (24%). This finding is in agreement with the results of several other bibliometric studies conducted in other medical fields including orthopedic surgery [68], anesthesia [59], and general surgery [60].

A distinctive characteristic of this analysis was that it included 10 evidence level-1 studies, including systematic reviews, meta-analyses, and randomized controlled trials. These findings do not coincide with the findings of several other bibliometric analyses performed on various specialties within dentistry and medicine [16,25,68,69,70]. Recently, these high evidence level studies have been performed and are securing high citations, despite only being published in recent years [71]. Such reports are useful for facilitating decision-making, directing practice, and advancing research, so a high number of such studies in the current study is not surprising and provides further proof of the maturation of the discipline [72].

This bibliometric analysis has several limitations. First, for a given research field, many factors may influence the citation count, including the age of the publication, journal of publication, the reputation of author, institution, and country of origin as well as the original language. Second, the analysis of self-citations and citations in textbooks and lectures was not performed. Moreover, the fact that some authors may be inclined to cite articles from a particular journal in which they intend to publish an article [73]. Third, the analysis of the contributing countries was based on the address of the corresponding author. A statistical bias may occur once the address of the corresponding author is changed [74]. Furthermore, for corresponding authors working in multiple institutions, we only considered the first institution.

## 4. Materials and Methods

### 4.1. Search Strategy

A total of 91 journals included in the category “Dentistry, Oral Surgery, and Medicine” in the database of the 2019 edition of the Journal Citation Report: Science Edition, a section of the Clarivate Analytics (https://www.jcr.clarivate.com) (accessed on 1 January 2021) were selected. An electronic literature search on Scopus (https://www.scopus.com) (accessed on 1 January 2021) database was performed on 1 January 2021. The journals *American Journal of Orthodontics*, now called the *American Journal of Orthodontics and Dentofacial Orthopedics*, the *International Journal of Oral Surgery*, now called as the *International Journal of Oral and Maxillofacial Surgery*, and *Critical Reviews in Oral Biology and Medicine*, now affiliated with the *Journal of Dental Research*, were also reviewed.

As the search strategy for each journal, the journal’s title was written in the source title’ section without any restriction of language, publication year, and study design of the article. Using the ‘documents’ tool of Scopus, the citation counts of all the articles published in all dentistry journals were identified.

### 4.2. Article Selection

According to the selected database, 336,381 articles were retrieved, out of which, the top 100 most-cited publications were further selected for this bibliometric analysis. The top 100 most-cited articles were selected and ranked based on their citation count. After ranking these articles, their cross-matching was performed with the citation data from Google Scholar to evaluate any fluctuation in citation counts.

### 4.3. Data Extraction and Bibliometric Variables

A total of 100 articles were included in this study, and their complete text was downloaded. The following bibliometric variables were extracted: publication title, citation count, current citation count (i.e., the total number of citation count collected by an article in 2020) [75], citation density (i.e., the total number of citation count/age of publication) [75], publication year, authorship, country of origin, study design, the field of interest, evidence level, and journal of publication.

Based on the study design, the articles were categorized as animal study, classification or tool for assessing the results, case-control study, cohort study, consensus report, in vitro study, letter to the editor, narrative review/expert opinion, new material or technique, randomized controlled trial, and systematic review/meta-analysis. Based on the field of interest, the articles were classified as adhesive restorations/dental materials, bone morphology/histology, behavior management, dental caries, endodontics, implantology, oral biology/morphology, oral pathology/medicine, oral radiology, orthodontics, oral hygiene, periodontology, pediatric dentistry, pain dysfunction/orofacial pain syndrome, regenerative dentistry, and saliva/biochemistry.

### 4.4. Data and Statistical Analysis

The Visualization of Similarities (VOSviewer) software (Centre for Science and Technology Studies, Leiden University, Leiden, The Netherlands) [76] was employed to visually analyze the registers separately, drawing a network of links among prominent authors, contributing countries, publishing journals, and author keywords to identify the strongest link of the net. The reason for selecting this software to draw and represent large networks from bibliometric information among other software, including Pajek or Gephi, is the remarkable display quality, the choice of demonstrating the density of links, and the probability of creating overlay maps adding data batches. Moreover, this software has been employed in several bibliometric analyses [75,77,78,79,80]. The characteristics are relevant for performing our bibliometric analysis.

Descriptive and bivariate analyses were performed using a statistical software package, i.e., IBM SPSS Statistics version 24.0 (IBM, Chicago, IL, USA). To assess the normality of the data, the Shapiro-Wilk test was conducted. Mean (standard deviation) or median (interquartile range) were calculated based on normality and distribution of data. To evaluate the median differences between the independent groups, the Kruskal–Wallis test was performed. Post hoc testing was performed to assess the median differences within each group. Any decrease or increase in the time-dependent trends was analyzed by performing the Mann–Kendall trend test. The Spearman-rank test was performed to assess the correlation between the publication count of the journal and the age of the journal. A value of *p* < 0.05 was considered statistically significant.

## 5. Conclusions

An appropriate selection of search engine and search strategy are extremely important to conduct a thorough bibliometric analysis. In this study, changing the search database resulted in several prominent differences when compared with the outcomes of a similar analysis published by Feijoo et al. [9] in 2014. The current study reported that narrative reviews/expert opinions related to periodontology having evidence level V were the most-cited articles in dentistry.

## Figures and Tables

**Table 1:** Contribution of authors to the top 100 most-cited articles in dentistry.

Author Name *	Number of Articles	Citation Count
Marx RE	7	8230
Löe H	4	12,668
Lekholm U	4	6654
Haffajee AD	4	5313
Socransky SS	4	4843
Albrektsson T	4	4658
De Munck J	4	3772
Genco RJ	4	3014
Brånemark PI	3	6140
Mehrotra B	3	3183
Ruggiero SL	3	3183
Lambrechts P	3	3156
Van Landuyt K	3	3049
Van Meerbeek B	3	3049
Yoshida Y	3	2620
Sjögren U	3	2444
Sundqvist G	3	2444
Lindhe J	3	2439
Zambon JJ	3	2144
Berglundh T	3	2112

* Due to a high number of contributing authors to the top 100 most-cited articles, it was not possible to mention all the authors in a table. Hence, the authors who contributed to ≥3 articles were included in the table.

**Table 2:** List of journals in which the top 100 most-cited articles were published.

Journal Name	JIF (2019) *	5-Year JIF *	No. of Articles	Citation Count
*J Dent Res*	4.914	5.844	17	17,836
*J Periodontol*	3.742	3.614	11	12,141
*J Clin Periodontol*	5.241	5.213	9	8461
*J Oral Maxillofac Surg*	1.642	2.020	8	8873
*Dent Mater*	4.495	5.386	7	6220
*J Endod*	3.118	3.380	5	3927
*Periodontol 2000*	7.718	8.888	4	3391
*Int J Oral Maxillofac Surg*	2.068	2.987	3	4200
*Oral Surg Oral Med Oral Pathol Radiol*	1.601	1.810	3	3345
*J Prosthet Dent*	2.444	2.727	3	2915
*Acta Odontol Scand*	1.573	1.785	2	8549
*Int J Oral Maxillofac Implants*	2.320	2.987	2	3996
*Commun Dent Oral Epidemiol*	2.135	2.558	2	2310
*J Oral Pathol Med*	2.495	2.330	2	2166
*Commun Dent Health*	0.679	1.140	2	2064
*J Am Dent Assoc*	2.803	2.950	2	1816
*Am J Orthod Dentofac Orthop*	1.960	2.405	2	1814
*Clinical Oral Implants Research*	3.723	4.044	2	1723
*Eur J Oral Sci*	2.220	2.225	2	1667
*Int Dent J*	2.038	1.863	1	1651
*Oral Oncol*	3.979	-	1	1585
*Oper Dent*	2.213	2.954	1	1248
*Int J Periodontics Restorative Dent*	1.513	1.739	1	968
*J Oral Fac Pain Headache*	1.260	2.421	1	941
*Implant Dent*	1.452	1.606	1	781
*Arch Oral Biol*	1.931	2.112	1	752
*J Can Dent Assoc*	1.200	0.917	1	735
*J Dent*	3.242	4.265	1	725
*Int Endod J*	3.801	3.418	1	721
*Int J Prosthod*	1.490	1.692	1	678
*J Dent Edu*	1.322	1.371	1	649

* Journal Citation Report (JCR) 2019. Abbreviation: JIF = Journal impact factor. Source for impact factor: https://www.jcr.clarivate.com (accessed on 5 January 2021).

**Table 3:** Distribution of fields of interest, study designs, and evidence levels of the top 100 most-cited articles.

Variable	Publications per	Citation Count	Median (min-max)	*p*-Value
**Field of Interest**				
Periodontology	26%	32,410	818.5 (638–4728)	*p* = 0.274
Adhesive Restorations	14%	11,915	724 (638–1560)	
Implantology	13%	15,592	838 (649–3341)	
Oral Medicine/Pathology	12%	12,785	927.5 (662–1798)	
Oral Hygiene	8%	10,643	1157.5 (717–1311)	
Endodontics	8%	5936	780 (656–883)	
Bone morphology/Histology	7%	6943	845 (692–1813)	
Oral Biology/Morphology	4%	5862	1450.5 (756–2517)	
Regenerative Dentistry (Stem cells)	2%	2228	1114 (979–1249)	
Orthodontics	2%	1814	907 (719–1095)	
Pain dysfunction/Orofacial pain syndrome	1%	941	941 (941)	
Saliva/Biochemistry	1%	917	917 (917)	
Behavior Management	1%	735	735 (735)	
Dental Radiology	1%	735	735 (735)	
**Study Design**				
Narrative review/Expert opinion	36%	34,628	831.5 (637–2517)	*p* = 0.145
Clinical trial	24%	34,296	952 (638–4602)	
Classification or tool for evaluating results	11%	14,072	1099 (703–2350),	
Systematic review/Meta-analysis	9%	6627	713 (664–845)	
In vitro study	7%	7561	808 (656–1813)	
Animal study	4%	4063	884.5 (831–1463)	
New material or technique	4%	3048	741.5 (655–910)	
Cohort study	2%	1879	939.5 (883–996)	
Letter to editor	1%	1798	1798 (1798)	
Consensus report	1%	767	767 (767)	
Randomized controlled trial	1%	717	717 (717)	

**Table 4:** Comparative analysis of the differences between the present study and Feijoo et al. [9].

Feijoo et al. [9]	Present Study
**Database Employment**	
Clarivate Analytics’ Web of Science (Benchmark)	Elsevier’s Scopus (Benchmark)
-	Google Scholar
-	
**Assessed Bibliometric Parameters**	
7	10
**Citation Count**	
Total citation count: 52,635 (WoS) - - Range of citation count: 326–2050 (WoS) - - Articles with ≥1000 citations: 4 Articles with ≥500 citations: 35	Total citation count: 113,482 (ES) 214,642 (GS) Range of citation count: 638 and 4728 (ES) 138 and 8281 (GS) Articles with ≥1000 citations: 33 Articles with ≥500 citations: 100
**Authorship**	
Articles with single author: 25 Articles with two authors: 18 Articles with more than 6 authors: 12 Leading author: Socransky SS (*n* = 9)	Articles with single author: 20 Articles with two authors: 27 Articles with more than 6 authors: 16 Leading author: Marx RE (*n* = 7)
**Publication Year**	
Decade with most publications: 1980s (26%)	Decade with most publications: 2000s (40%)
**Field of Interest**	
1st = Periodontology (43%) 2nd = Implantology (11%) 3rd = Adhesive restorations (8%)	1st = Periodontology (26%) 2nd = Adhesive restorations (14%) 3rd = Implantology (13%)
**Study Design**	
1st = Cases series (22%) 2nd = Narrative review/expert opinion (19%) 3rd = Classifications or tools for evaluating results (13%)	1st = Narrative review/expert opinion (36%) 2nd = Clinical trial (24%) 3rd = Classifications or tools for evaluating results (11%)
**Evidence Level**	
EL V = 54%	EL V = 64%
**Journal of Publication**	
Total number of journals: 22	Total number of journals: 32
1st = *Journal of Clinical Periodontology* (20%) 2nd = *Journal of Periodontology* (18%) 3rd = *Journal of Dental Research* (16%)	1st = *Journal of Dental Research* (17%) 2nd = *Journal of Periodontology* (11%) 3rd = *Journal of Clinical Periodontology* (9%)

Abbreviation: EL V = evidence level Five; ES = Elsevier’s Scopus; GS = Google Scholar; WoS = Web of Science.

## Supplementary Materials

The following are available online at https://www.mdpi.com/2227-9032/9/3/356/s1, Table S1: The list of the top 100 most-cited articles published in the dentistry.
