# Supplementary material for: The Top 100 Most Cited Articles Published in Dentistry: 2020 Update
Source: Healthcare (Basel). 2021 Mar 21;9(3):356. doi: 10.3390/healthcare9030356 (PMC8003932; doi:10.3390/healthcare9030356)
Supplement: Supplementary file 1 [file healthcare-09-00356-s001.pdf]

Table 1. The list of the top 100 most-cited articles published in the dentistry.

Sr No.	Article title	Citation count (ES)	Citation count (GS)	Citation density	CCI 2020
1	Silness J, Löe H. Periodontal disease in pregnancy II. Correlation between oral hygiene and periodontal condition. *Acta Odontol Scand*. 1964;22:121-135.	4728	8281	84	269
2	Löe H, Silness J. Periodontal disease in pregnancy I. Prevalence and severity. *Acta Odontol Scand*. 1963;21:533-551.	4062	7873	71	232
3	Adell R, Lekholm U, Rockler B, Brånemark P-I. A 15-year study of osseointegrated implants in the treatment of the edentulous jaw. *Int J Oral Surg*. 1981;10:387-416.	3392	6257	117	96
4	Socransky S, Haffajee A, Cugini M, Smith C, Kent Jr R. Microbial complexes in subgingival plaque. *J Clin Periodontol*. 1998;25:134-144.	2633	4683	120	217
5	Birkedal-Hansen H, Moore W, Bodden M, Windsor L, Birkedal-Hansen B, DeCarlo A, Engler J. Matrix metalloproteinases: a review. *Crit Rev Oral Biol Med*. 1993;4:197-250.	2529	3620	94	36
6	Albrektsson T, Zarb G, Worthington P, Eriksson A. The long-term efficacy of currently used dental implants: a review and proposed criteria of success. *Int J Oral Maxillofac Implants*. 1986;1:11-25.	2417	5233	71	115
7	Löe H. The gingival index, the plaque index and the retention index systems. *J Periodontol*. 1967;38:610-616.	2342	4136	44	168
8	Löe H, Theilade E, Jensen SB. Experimental gingivitis in man. *J Periodontol*. 1965;36:177-187.	1904	4376	35	80
9	Marx RE, Carlson ER, Eichstaedt RM, Schimmele SR, Strauss JE, Georgeff KR. Platelet-rich plasma: growth factor enhancement for bone grafts. *Oral Surg Oral Med Oral Pathol Oral Radiol Endodontol*. 1998;85:638-646.	1870	3663	85	111
10	Guo Sa, DiPietro LA. Factors affecting wound healing. *J Dent Res*. 2010;89:219-229.	1859	3384	186	345
11	Marx RE. Pamidronate (Aredia) and zoledronate (Zometa) induced avascular necrosis of the jaws: a growing epidemic. *J Oral Maxillofac Surg*. 2003;61:1115-1117.	1852	3249	109	107
12	Ainamo J, Bay I. Problems and proposals for recording gingivitis and plaque. *Int Dent J*. 1975;25:229.	1735	3066	38	160
13	Warnakulasuriya S. Global epidemiology of oral and oropharyngeal cancer. *Oral Oncol*. 2009;45:309-316.	1700	2841	155	234
14	Adell R, Eriksson B, Lekholm U, Brånemark P-I, Jemt T. A long-term follow-up study of osseointegrated implants in the treatment of totally edentulous jaws. *Int J Oral Maxillofac Implants*. 1990;5.	1661	2982	55	28
15	Buonocore MG. A simple method of increasing the adhesion of acrylic filling materials to enamel surfaces. *J Dent Res*. 1955;34:849-853.	1588	4476	24	45
16	Le Guéhennec L, Soueidan A, Layrolle P, Amouriq Y. Surface treatments of titanium dental implants for rapid osseointegration. *Dent Mater*. 2007;23:844-854.	1560	2426	120	148

17	Ruggiero SL, Mehrotra B, Rosenberg TJ, Engroff SL. Osteonecrosis of the jaws associated with the use of bisphosphonates: a review of 63 cases. *J Oral Maxillofac Surg*. 2004;62:527-534.	1509	2555	94	40
18	Donath K, Breuner G. A method for the study of undecalcified bones and teeth with attached soft tissues: The Säge-Schliff (sawing and grinding) Technique. *J Oral Pathol Med*. 1982;11:318-326.	1480	2040	39	44
19	O'Leary TJ, Drake RB, Naylor JE. The plaque control record. *J Periodontol*. 1972;43:38-38.	1371	2431	29	150
20	Slade GD, Spencer AJ. Development and evaluation of the oral health impact profile. *Commun Dent Health*. 1994;11:3.	1353	2620	52	127
21	Gronthos S, Brahim J, Li W, Fisher L, Cherman N, Boyde A, DenBesten P, Robey PG, Shi S. Stem cell properties of human dental pulp stem cells. *J Dent Res*. 2002;81:531-535.	1299	2305	72	90
22	Van Meerbeek B, De Munck J, Yoshida Y, Inoue S, Vargas M, Vijay P, Van Landuyt K, Lambrechts P, Vanherle G. Adhesion to enamel and dentin: current status and future challenges. *Oper Dent*. 2003;28:215-35.	1294	2641	76	69
23	Petersen PE. The World Oral Health Report 2003: continuous improvement of oral health in the 21st century–the approach of the WHO Global Oral Health Programme. *Commun Dent Oral Epidemiol*. 2003;31:3-24.	1291	3873	76	126
24	Moorrees CF, Fanning EA, Hunt Jr EE. Age variation of formation stages for ten permanent teeth. *J Dent Res*. 1963;42:1490-1502.	1245	2404	22	66
25	Marx RE. Platelet-rich plasma: evidence to support its use. *J Oral Maxillofac Surg*. 2004;62:489-496.	1196	2374	75	89
26	De Munck Jd, Van Landuyt K, Peumans M, Poitevin A, Lambrechts P, Braem M, Van Meerbeek B. A critical review of the durability of adhesion to tooth tissue: methods and results. *J Dent Res*. 2005;84:118-132.	1196	2409	82	73
27	Branemark P-I. Osseointegration and its experimental background. *J Prosthet Dent*. 1983;50:399-410.	1188	2737	32	65
28	Marx RE, Sawatari Y, Fortin M, Broumand V. Bisphosphonate-induced exposed bone (osteonecrosis/osteopetrosis) of the jaws: risk factors, recognition, prevention, and treatment. *J Oral Maxillofac Surg*. 2005;63:1567-1575.	1166	2039	78	46
29	Slade GD. Derivation and validation of a short-form oral health impact profile. *Commun Dent Oral Epidemiol*. 1997;25:284-290.	1165	2311	51	163
30	Greene JG, Vermillion JR. The simplified oral hygiene index. *J Am Dent Assoc*. 1964;68:7-13.	1162	2723	21	120
31	Huang G-J, Gronthos S, Shi S. Mesenchymal stem cells derived from dental tissues vs. those from other sources: their biology and role in regenerative medicine. *J Dent Res*. 2009;88:792-806.	1034	1721	94	98
32	Haffajee AD. Microbial etiological agents of destructive periodontal diseases. *Periodontol 2000*. 1994;5:78-111.	1132	2307	44	31
33	Denry I, Kelly JR. State of the art of zirconia for dental applications. *Dent Mater*. 2008;24:299-307.	1132	2066	94	160

34	Houston W. The analysis of errors in orthodontic measurements. *Am J Orthod*. 1983;83:382-390.	1116	2055	30	49
35	Schiffman E, Ohrbach R, Truelove E, Look J, Anderson G, Goulet J-P, List T, Svensson P. Diagnostic criteria for temporomandibular disorders (DC/TMD) for clinical and research applications: recommendations of the International RDC/TMD Consortium Network and Orofacial Pain Special Interest Group. *J Oral Fac Pain Headache*. 2014;28:6.	1087	1772	181	299
36	Schropp L, Wenzel A, Kostopoulos L, Karring T. Bone healing and soft tissue contour changes following single-tooth extraction: a clinical and radiographic 12-month prospective study. *Int J Periodont Restor Dent*. 2003;23.	1019	2149	60	96
37	Beck J, Garcia R, Heiss G, Vokonas PS, Offenbacher S. Periodontal disease and cardiovascular disease. *J Periodontol*. 1996;67:1123-1137.	1016	2008	42	37
38	Ruggiero SL, Dodson TB, Fantasia J, Goodday R, Aghaloo T, Mehrotra B, O'Ryan F. American Association of Oral and Maxillofacial Surgeons position paper on medication-related osteonecrosis of the jaw— 2014 update. *J Oral Maxillofac Surg*. 2014;72:1938-1956.	1001	1509	167	244
39	Turesky S, Gilmore ND, Glickman I. Reduced plaque formation by the chloromethyl analogue of victamine C. *J Periodontol*. 1970;41:41-43.	997	1512	20	57
40	Humphrey SP, Williamson RT. A review of saliva: normal composition, flow, and function. *J Prosthet Dent*. 2001;85:162-169.	989	1897	52	149
41	Araújo MG, Lindhe J. Dimensional ridge alterations following tooth extraction. An experimental study in the dog. *J Clin Periodontol*. 2005;32:212-218.	980	2051	65	105
42	Buser D, Mericske-stern R, Pierre Bernard JP, Behneke A, Behneke N, Hirt HP, Belser UC, Lang NP. Long-term evaluation of non-submerged ITI implants. Part 1: 8-year life table analysis of a prospective multi-center study with 2359 implants. *Clin Oral Implants Res*. 1997;8:161-172.	952	1865	41	26
43	Zehnder M. Root canal irrigants. *J Endod*. 2006;32:389-398.	938	2101	67	114
44	Socransky SS, Haffajee AD. Periodontal microbial ecology. *Periodontol 2000*. 2005;38:135-187.	935	1822	62	79
45	Feilzer A, De Gee A, Davidson C. Setting stress in composite resin in relation to configuration of the restoration. *J Dent Res*. 1987;66:1636-1639.	931	1642	28	23
46	Eke PI, Dye B, Wei L, Thornton-Evans G, Genco R. Prevalence of periodontitis in adults in the United States: 2009 and 2010. *J Dent Res*. 2012;91:914-920.	930	1611	116	109
47	Kornman KS, Crane A, Wang HY, Giovlne FSd, Newman MG, Pirk FW, Wilson Jr TG, Higginbottom FL, Duff GW. The interleukin-1 genotype as a severity factor in adult periodontal disease. *J Clin Periodontol*. 1997;24:72-77.	896	1585	39	20
48	Sjögren U, Hägglund B, Sundqvist G, Wing K. Factors affecting the long-term results of endodontic treatment. *J Endod*. 1990;16:498-504.	918	2191	31	63
49	Ferracane JL. Resin composite—state of the art. *Dent Mater*. 2011;27:29-38.	918	1838	102	138

50	Cawood J, Howell R. A classification of the edentulous jaws. *Int J Oral Maxillofac Surg*. 1988;17:232-236.	898	1581	28	55
51	Sundqvist G, Figdor D, Persson S, Sjögren U. Microbiologic analysis of teeth with failed endodontic treatment and the outcome of conservative re-treatment. *Oral Surgery, Oral Medicine, Oral Pathol Oral Radiol Endodontol*. 1998;85:86-93.	869	1971	40	36
52	Eriksson A, Albrektsson T. Temperature threshold levels for heat-induced bone tissue injury: a vital-microscopic study in the rabbit. *J Prosthet Dent*. 1983;50:101-107.	864	1505	23	56
53	Esposito M, Hirsch J-M, Lekholm U, Thomsen P. Biological factors contributing to failures of osseointegrated oral implants.(II). Etiopathogenesis. *Eur J Oral Sci*. 1998;106:721.	861	1617	39	33
54	Buser D, Broggini N, Wieland M, Schenk R, Denzer A, Cochran D, Hoffmann B, Lussi A, Steinemann S. Enhanced bone apposition to a chemically modified SLA titanium surface. *J Dent Res*. 2004;83:529-533.	860	1326	54	60
55	Jaffin RA, Berman CL. The excessive loss of Branemark fixtures in type IV bone: a 5-year analysis. *J Periodontol*. 1991;62:2-4.	848	1585	29	22
56	Offenbacher S, Katz V, Fertik G, Collins J, Boyd D, Maynor G, McKaig R, Beck J. Periodontal infection as a possible risk factor for preterm low birth weight. *J Periodontol*. 1996;67:1103-1113.	846	2000	35	30
57	Esposito M, Hirsch JM, Lekholm U, Thomsen P. Biological factors contributing to failures of osseointegrated oral implants,(I). Success criteria and epidemiology. *Eur J Oral Sci*. 1998;106:527-551.	841	1591	38	35
58	Page RC, Eke PI. Case definitions for use in population-based surveillance of periodontitis. *J Periodontol*. 2007;78:1387-1399.	835	1301	64	94
59	Sodek J, Ganss B, McKee M. Osteopontin. *Crit Rev Oral Biol Med*. 2000;11:279-303.	832	1262	42	35
60	Wennerberg A, Albrektsson T. Effects of titanium surface topography on bone integration: a systematic review. *Clin Oral Implants Res*. 2009;20:172-184.	831	1289	76	81
61	Torabinejad M, Hong C, McDonald F, Ford TP. Physical and chemical properties of a new root-end filling material. *J Endod*. 1995;21:349-353.	829	2013	33	48
62	Ainamo J. Development of the World Health Organization (WHO) community periodontal index of treatment needs (CPITN). *Int Dent J* 1982;32:281-291.	825	1694	22	38
63	Ruggiero SL, Dodson TB, Assael LA, Landesberg R, Marx RE, Mehrotra B. American Association of Oral and Maxillofacial Surgeons position paper on bisphosphonate-related osteonecrosis of the jaws—2009 update. *J Oral Maxillofac Surg*. 2009;67:2-12.	822	1206	75	42
64	Marx RE. Platelet-rich plasma (PRP): what is PRP and what is not PRP? *Implant Dent*. 2001;10:225-228.	818	1780	43	71
65	Lindhe J, Meyle J, Berglundh T, Claffey N, De Bruyn H, Heitz-Mayfield N, Karoussis I, Könönen E, Mombelli A, Renvert S, van Winkelhoff A, Winkel E, Zitzmann N. Peri-implant diseases: consensus	812	1600	68	99

	report of the sixth European workshop on periodontology. *J Clin Periodontol*. 2008;35:282-285.				
66	Bollenl CM, Lambrechts P, Quirynen M. Comparison of surface roughness of oral hard materials to the threshold surface roughness for bacterial plaque retention: a review of the literature. *Dent Mater*. 1997;13:258-269.	811	1497	35	99
67	Socransky SS, Haffajee AD. The bacterial etiology of destructive periodontal disease: current concepts. *J Periodontol*. 1992;63:322-331.	806	1685	29	18
68	Locker D. Measuring oral health: a conceptual framework. *Commun Dent Health*. 1988;5:3-18.	782	1595	24	71
69	Grossi SG, Zambon JJ, Ho AW, Koch G, Dunford RG, Machtei EE, Norderyd OM, Genco RJ. Assessment of risk for periodontal disease. I. Risk indicators for attachment loss. *J Periodontol*. 1994;65:260-267.	769	1572	30	20
70	Manicone PF, Iommetti PR, Raffaelli L. An overview of zirconia ceramics: basic properties and clinical applications. *J Dent*. 2007;35:819-826.	768	1447	59	80
71	Scarfe WC, Farman AG, Sukovic P. Clinical applications of cone-beam computed tomography in dental practice. *J Can Dent Assoc*. 2006;72:75.	761	1948	54	64
72	Gold OG, Jordan H, Van Houte J. A selective medium for Streptococcus mutans. *Arch Oral Biol*. 1973;18:1357-1364.	759	1218	16	13
73	Nyman S, Lindhe J, Karring T, Rylander H. New attachment following surgical treatment of human periodontal disease. *J clin periodontol*. 1982;9:290-296.	757	1662	20	16
74	Marx RE. Osteoradionecrosis: a new concept of its pathophysiology. *J Oral Maxillofac Surg*. 1983;41:283-288.	756	1251	20	54
75	Warnakulasuriya S, Johnson NW, Van der Waal I. Nomenclature and classification of potentially malignant disorders of the oral mucosa. *J Oral Pathol Med*. 2007;36:575-580.	754	1482	58	93
76	Haraszthy V, Zambon J, Trevisan M, Zeid M, Genco R. Identification of periodontal pathogens in atheromatous plaques. *J Periodontol*. 2000;71:1554-1560.	752	1408	38	30
77	Moore W, Moore LV. The bacteria of periodontal diseases. *Periodontol 2000*. 1994;5:66-77.	751	1405	29	28
78	Corah NL. Development of a dental anxiety scale. *J Dent Res*. 1969;48:596-596.	747	1626	15	26
79	Dohan DM, Choukroun J, Diss A, Dohan SL, Dohan AJ, Mouhyi J, Gogly B. Platelet-rich fibrin (PRF): a second-generation platelet concentrate. Part I: technological concepts and evolution. *Oral Surg Oral Med Oral Pathol Oral Radiol Endodontol*. 2006;101:e37-e44.	747	1605	53	119
80	Breschi L, Mazzoni A, Ruggeri A, Cadenaro M, Di Lenarda R, Dorigo EDS. Dental adhesion review: aging and stability of the bonded interface. *Dent Mater*. 2008;24:90-101.	746	1413	62	65
81	Yoshida Y, Nagakane K, Fukuda R, Nakayama Y, Okazaki M, Shintani H, Inoue S, Tagawa Y, Suzuki K, De Munck J. Comparative study on adhesive performance of functional monomers. *J Dent Res*. 2004;83:454-458.	745	1246	47	58

82	Årtun J, Bergland S. Clinical trials with crystal growth conditioning as an alternative to acid-etch enamel pretreatment. *Am J Orthod*. 1984;85:333-340.	738	1311	21	58
83	Sjögren U, Figdor D, Persson S, Sundqvist G. Influence of infection at the time of root filling on the outcome of endodontic treatment of teeth with apical periodontitis. *Int Endod J*. 1997;30:297-306.	737	1613	32	35
84	Quigley GA, Hein JW. Comparative cleansing efficiency of manual and power brushing. *J Am Dent Assoc*. 1962;65:26-29.	737	1216	13	33
85	Ferracane JL. Hygroscopic and hydrolytic effects in dental polymer networks. *Dent Mater*. 2006;22:211-222.	732	1263	52	79
86	Torabinejad M, Chivian N. Clinical applications of mineral trioxide aggregate. *J Endod*. 1999;25:197-205.	731	1818	35	33
87	Van Meerbeek B, Yoshihara K, Yoshida Y, Mine A, De Munck J, Van Landuyt K. State of the art of self-etch adhesives. *Dent Mater*. 2011;27:17-28.	703	1282	78	106
88	Albrektsson T, Wennerberg A. Oral implant surfaces: Part 1--review focusing on topographic and chemical properties of different surfaces and in vivo responses to them. *Int J Prosthod*. 2004;17.	700	1156	44	44
89	Berglundh T, Persson L, Klinge B. A systematic review of the incidence of biological and technical complications in implant dentistry reported in prospective longitudinal studies of at least 5 years. *J Clin Periodontol*. 2002;29:197-212.	698	1331	39	40
90	Zitzmann NU, Berglundh T. Definition and prevalence of peri-implant diseases. *J Clin Periodontol*. 2008;35:286-291.	689	1355	57	59
91	Peters OA. Current challenges and concepts in the preparation of root canal systems: a review. *J Endod*. 2004;30:559-567.	683	1564	43	64
92	Socransky SS. Dental biofilms: difficult therapeutic targets. *Periodontol 2000*. 2002;28:12-55.	682	1475	38	48
93	Pashley DH, Tay F, Yiu C, Hashimoto M, Breschi L, Carvalho R, Ito S. Collagen degradation by host-derived enzymes during aging. *J Dent Res*. 2004;83:216-221.	679	1158	42	42
94	Quirynen M, Bollen C. The influence of surface roughness and surface-free energy on supra-and subgingival plaque formation in man: A review of the literature. *J Clin Periodontol*. 1995;22:1-14.	669	1206	27	60
95	Davies JE. Understanding peri-implant endosseous healing. *J Dent Edu*. 2003;67:932-949.	667	1209	39	43
96	Advisory T. American Association of Oral and Maxillofacial Surgeons position paper on bisphosphonate-related osteonecrosis of the jaws. *J Oral Maxillofac Surg*. 2007;65:369-376.	668	138	51	14
97	Davidson C, De Gee A, Feilzer A. The competition between the composite-dentin bond strength and the polymerization contraction stress. *J Dent Res*. 1984;63:1396-1399.	660	1200	18	12
98	Johnston W, Kao E. Assessment of appearance match by visual observation and clinical colorimetry. *J Dent Res*. 1989;68:819-822.	652	1216	21	34

99	Zambon JJ. Actinobacillus actinomycetemcomitans in human periodontal disease. *J Clin Periodontol*. 1985;12:1-20.	649	1030	19	10
100	Slots J, Genco RJ. Black-pigmented Bacteroides species, Capnocytophaga species, and Actinobacillus actinomycetemcomitans in human periodontal disease: virulence factors in colonization, survival, and tissue destruction. *J Dent Res*. 1984;63:412-421.	638	1010	18	4
